# A survey on autonomous environmental monitoring approaches: towards unifying active sensing and reinforcement learning

**DOI:** 10.3389/frobt.2024.1336612

**Published:** 2024-03-12

**Authors:** David Mansfield, Allahyar Montazeri

**Affiliations:** Lancaster University, School of Engineering, Lancaster, United Kingdom

**Keywords:** reinforcement learning, environmental monitoring, active sensing, deep learning, robotics, multi-agent

## Abstract

The environmental pollution caused by various sources has escalated the climate crisis making the need to establish reliable, intelligent, and persistent environmental monitoring solutions more crucial than ever. Mobile sensing systems are a popular platform due to their cost-effectiveness and adaptability. However, in practice, operation environments demand highly intelligent and robust systems that can cope with an environment’s changing dynamics. To achieve this reinforcement learning has become a popular tool as it facilitates the training of intelligent and robust sensing agents that can handle unknown and extreme conditions. In this paper, a framework that formulates active sensing as a reinforcement learning problem is proposed. This framework allows unification with multiple essential environmental monitoring tasks and algorithms such as coverage, patrolling, source seeking, exploration and search and rescue. The unified framework represents a step towards bridging the divide between theoretical advancements in reinforcement learning and real-world applications in environmental monitoring. A critical review of the literature in this field is carried out and it is found that despite the potential of reinforcement learning for environmental active sensing applications there is still a lack of practical implementation and most work remains in the simulation phase. It is also noted that despite the consensus that, multi-agent systems are crucial to fully realize the potential of active sensing there is a lack of research in this area.

## 1 Introduction

The fields of Artificial Intelligence (AI) and robotics are accelerating the world toward its next technological revolution. Advances in both of these fields bring about new and exciting technologies that can be used to help tackle many of the challenges we face in life on planet Earth. One of the most pressing issues is climate change for which environmental monitoring (EM) plays a vital role in understanding and mitigating the impact of both natural and human activity that contribute to this growing issue. Before we can implement *in situ* solutions, we first need a deep understanding of each specific issue which in many cases requires scientists to collect much more empirical data at each site of interest, as in reality, no two sites are exactly the same. For many natural processes, over which we do not have direct control, forecasting is an invaluable practice. However, each case is unique and at many of the sites where forecasting would be beneficial researchers do not have enough consistent knowledge or data to make accurate predictions. A persistent EM system is paramount to the development of accurate foresight in these situations. However, traditional monitoring methods are often expensive, slow, dangerous or inefficient when compared to the full potential of an intelligent autonomous system.

Simply, EM is the task of making observations of an environmental phenomenon. In the literature EM is a blanket term which refers to many different applications such as pollution monitoring Alvear et al. (2017), boundary tracking Jin and Bertozzi (2007), search and rescue Waharte and Trigoni (2010), volcanology James et al. (2020), subterranean mapping Neumann et al. (2014) and many more similar applications. This paper also touches on other fields such as agriculture Duckett et al. (2018) and nuclear decommissioning Martin et al. (2016), wherein EM technology plays a vital role in various aspects of their operation. In all of these applications environmental phenomena often occur over large physical spaces, vary on massive scales and are largely unpredictable meaning they require persistent or periodic monitoring. Due to these complex and changing environments, it is necessary to design robots that can adapt their behaviour in real-time to fit the evolving environment according to their sensed data; this process is called active sensing Yang et al. (2016). In this paper, we discuss the emerging sub-category of active sensing concerned with monitoring environmental phenomena which will be referred to henceforth as *active environmental monitoring* (active EM).

**FIGURE 1:**
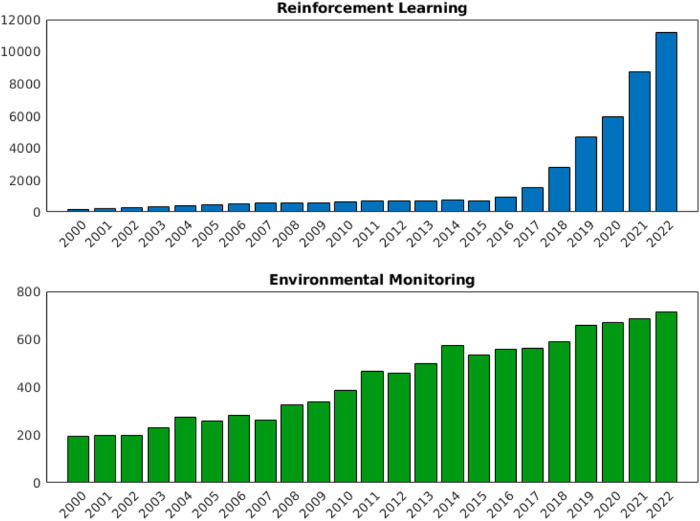
Number of publications with ‘RL’ and ‘EM’ in the title. Scraped from Google Scholar Strobel, (2018).

Active EM entails complex agent behaviours to achieve diverse goals, necessitating real-time adaptability and decision-making capabilities. This behaviour requirement lends itself naturally to machine learning since hard-coding these required behaviours can be complex Kober et al. (2013). Reinforcement Learning (RL) and Deep Reinforcement Learning (DRL) is a branch of machine learning that involves training agents to make optimal decisions by interacting with the environment. This process includes trial-and-error interactions aimed at maximizing rewards or achieving specific goals, leading to the development of an optimal policy for decision-making across various scenarios Mnih et al. (2015). For example, a system that has to operate in outdoor environments may be subject to multiple lighting levels, extreme weather conditions, and large areas of operation. RL has been demonstrated as an effective solution to allow robots to navigate under such varying conditions Maciel-Pearson et al. (2019). The increasing popularity of both RL and EM are demonstrated in Figure 1, evidencing the growing importance of research and development in both fields. However, not all the literature that involves EM systems is released under the term ‘environmental monitoring’ and standardizing this will help research accelerate.

In this review paper, a comprehensive overview of the application of RL to EM problems is provided. Upon reviewing the literature it was found that there are numerous trends in RL state representation across different EM applications. For example, it is common to represent the environment as a grid of coloured “tiles” that encode information about a spatial area Padrao et al. (2022); Luis et al. (2020). Accordingly, an alternative classification of RL algorithms in terms of continuous or discrete state and action spaces is included. This allows one to pick an algorithm based on the constraints of common EM problems. It is also shown by means of a unified formulation that the nature of RL and active sensing problems are complementary. In the proposed framework, both problems are described by a Decentralized Partially Observable Markov Decision Process (Dec-POMDP) which facilitates both single and multi-agent systems. Despite the synergy between these two problems and the growing body of research in both fields, it is found that there is a lack of practical implementation, environment-realistic simulation environments and research into multi-agent reinforcement learning (MARL) approaches. This paper provides researchers with a condensed view of common approaches to the application of RL for active EM and unifies them under a suitable framework.

## 2 Reinforcement learning for active environmental monitoring

### 2.1 Previous surveys

There are a number of previous surveys covering the application of RL to robotics through which their compatibility is well discussed. Kober et al. (2013) is a 2014 survey on the application of RL to robotic platforms. It gives a comprehensive explanation of how RL can be used in robotic research and highlights the challenges in the field. Azar et al. (2021) looks at the application of DRL to Unmanned Aerial Vehicles (UAVs). The authors note some open EM applications and their associated problems where RL and DRL can be Utilized. Lapeyrolerie et al. (2022) discusses how RL is suited to conservation applications and provides a good background that helps justify further research and the joining of these two fields. Canese et al. (2021) reviews the current challenges and applications of Multi-Agent Reinforcement Learning (MARL), again noting how developments in these systems would be well suited to EM problems. Also in the domain of MARL, Zhu et al. (2022) discusses communication in MARL systems and Oroojlooy and Hajinezhad (2023) reviews cooperative MARL, offering a novel classification system for how these systems are designed and trained. While much of the previous work highlights the potential of robotic platforms trained with RL for application to EM, they do not discuss the topic in great detail or highlight the current specific challenges that EM brings.

**FIGURE 2:**
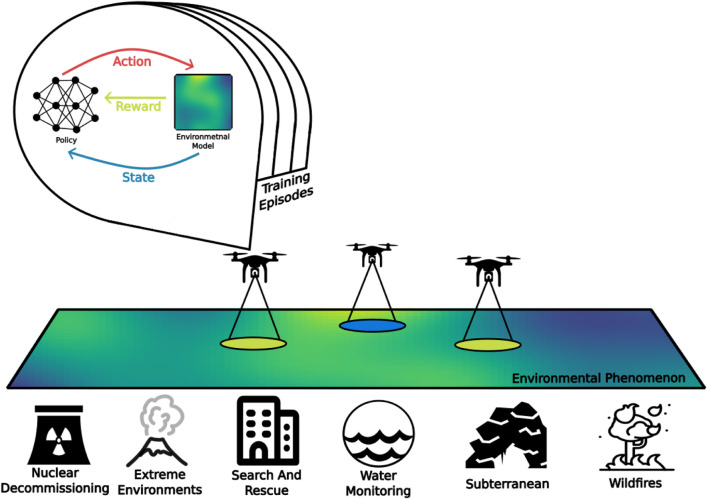
A general environmental monitoring problem, showing a model-based deep reinforcement learning trained UAV system.

Complementary to the previous surveys, this paper brings the following contributions: RL for robotic platforms is framed as an active EM problem, allowing for a clear picture of how RL can benefit such systems. This is done with a classification of RL by mission requirements rather than by algorithmic properties which is more suited for the research into active EM. It is also shown that active EM problems can be framed as a general active sensing problem and they are unified with RL problems under a single framework. In fact, the idea of *active environmental monitoring* generalizes the current approaches under a single framework by which state-of-the-art implementations are presented. A general RL-based active EM system is visualized in Figure 2. Finally, the challenges of practical implementation and open questions in the context of active EM problems are discussed.

### 2.2 Reinforcement learning terminologies

**FIGURE 3:**
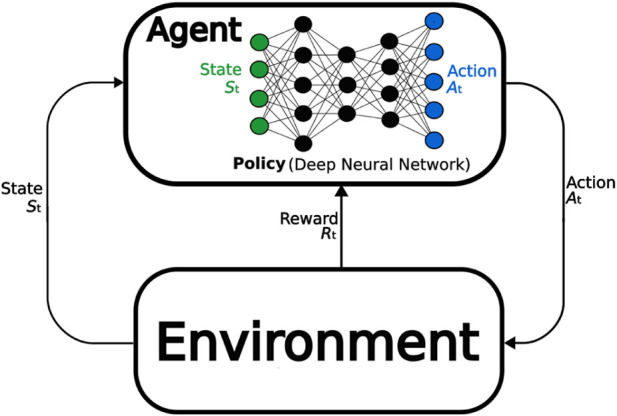
Deep RL visualized. In standard RL the policy is represented by other means than a deep neural network.

Reinforcement Learning (RL) is the process of training an *agent* to learn a *policy* that picks *actions* based on an *observation* of the *environment*. Sequences of individual actions should accumulate to a desired overall behaviour. The environment is represented and described by a *state*. Effective state-actions pairs are reinforced by a *reward*. The goal of the agent is to maximize the long-term (collective) reward by updating its policy. The goal of the policy is essentially to map state-action pairs to rewards, allowing us to choose the most effective action for a given state. RL can use various function approximates to represent the policy, including linear models or decision trees. This paper is also concerned with DRL. The defining difference between RL and DRL is that the policy in DRL is represented by a neural network. This is illustrated in Figure 3.

Different environments and different learning agents permit different actions. All of the actions that are valid for a given environment make up the *action space*. In a given state, the agent can choose any action in the action space. Actions are chosen either according to a policy or an exploration strategy. Action spaces can be continuous or discrete. Continuous action spaces can be used for tasks such as sending commands directly to robot actuators, whereas discrete action spaces are generally more abstract and involve more high-level commands such as, ‘move forward’ or ‘turn around’. Having an overly complex action space can lead to difficulties in training and the learning algorithm may not converge to an optimal policy. On the other hand, action spaces that are too simple may result in sub-optimal and limited behaviour.

In general RL and DRL algorithms are divided into model-based and model-free approaches. Model-based approaches have direct access to a learned or given model of the environment whereas model-free agents do not. Model-based techniques give an agent more information on the next optimal action to take, meaning that agents can quickly learn policies and make projections of future rewards. However, this is only true if the model of the environment is accurate. In lots of cases, the environments are complex and predicting them accurately is a difficult task in and of itself. That being said, model-based algorithms that have access to a strong environmental model can give state-of-the-art results and show a substantial improvement in sample efficiency Silver et al. (2017); Kaiser et al. (2020). However, model mismatch can have some detrimental consequences. For instance, the agent may learn to exploit the bias or inaccuracies in the model causing poor performance in the real-world environment. In cases where a reliable model is not present then model-free algorithms perform better Lapeyrolerie et al. (2022). RL algorithms can also be classified as on-policy where actions are chosen from the same policy used during evaluation Schulman et al. (2017) or off-policy algorithms where agents sample their actions from a separate policy Jang et al. (2019); Lillicrap et al. (2019). Algorithms can also be value-based or policy gradient-based. Value-based algorithms use a value function to quantify the ‘goodness’ or value of taking a certain action in a certain state. The most well-known family of value-based algorithms is the Q-learning approach Watkins and Dayan (1992). Instead, in policy-gradient methods, the agents perform gradient ascent on the expected reward function to find the optimal policy. There is a class of RL algorithms called Actor-Critic methods that combine different aspects of both value-based and policy-gradient-based RL algorithms. This class of algorithms consist of two main components: the actor, which is responsible for taking actions and learning a policy that maximizes reward, and the critic, which is responsible for estimating the value of taking an action in a given state. This helps the actor to make more informed decisions. Actor-critic methods have been used to produce some very promising results Mnih et al. (2016); Haarnoja et al. (2018).

**FIGURE 4:**
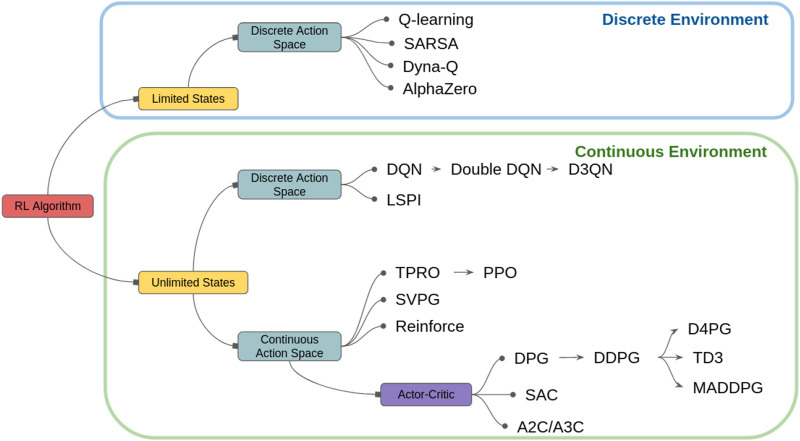
RL Classification by environment type AlMahamid and Grolinger (2021).

Generally speaking, various RL and DRL algorithms are reviewed and discussed in depth in the literature and an interested reader is referred to these papers for further discussions Lapeyrolerie et al. (2022); Kober et al. (2013); AlMahamid and Grolinger (2021). However, in the context of EM, the active sensing objectives are different for each application and are usually partially constrained according to the operating environment. Therefore, it would be more beneficial to classify the algorithms for EM applications based on their environment and action spaces. For example, we may need to train an agent to scan a given area. Depending on the mission objectives, we can decide to either abstract this area into smaller sub-areas allowing us to use a limited and discrete state and action space, or we can choose to predict a continuous model of the region of interest for which we need a continuous learning environment. A classification of standard and state-of-the-art RL algorithms used for EM applications is illustrated in Figure 4.

### 2.3 Reinforcement learning for single-agent environmental monitoring systems

Hard-coding complex and adaptive behaviours for robotic systems can often be a very difficult task, this is compounded by the fact that robots also often have non-linear dynamics and operate in complex environments whilst under considerable amounts of uncertainties and constraints. Unlike traditional programming techniques, RL allows engineers to take a more end-to-end approach when developing control and navigation algorithms. In this approach, the desired behaviour of a learning agent is described by designing a suitable reward function. The design of this reward function, a process which is called reward shaping, is much more high-level than programming the behaviours directly.

Reward functions should describe the goals of a system rather than specific behaviour. Under a well-designed reward function and a suitable RL algorithm, an agent can learn to behave optimally. A general reward function might take the form:

$$R=R_{\mathrm{goal}}+R_{\mathrm{penalty}},$$ (1)

where *R*_*goal*_ is a positive scalar that is given to an agent for actions that are productive to the overall goal of the system and *R*_*penalty*_ is a negative scalar that penalizes the robot for undesirable actions that are counterproductive to the goal. For example, Consider designing a reward function to teach a robot to follow a line on the x-axis to reach a goal.

$$R_{\mathrm{goal}}=\frac{1}{D}+\alpha v_{x},$$ (2)

where *D* is the distance to the goal, ***v***_***x***_ is the agent’s velocity in the x-direction and *α* is a constant controlling the contribution of velocity to the reward. *HereR*_*goal*_ rewards the robot for moving towards the goal at a higher velocity. To accompany this we might have:

$$R_{\mathrm{penalty}}=-|y{|}^{2}$$ (3)

where *y* is the robot’s y coordinate. Here, *R*_*penalty*_ increases with the square of the distance from the line, and thus encourages the robot to stay on the x-axis by administering a negative reward (penalty). Since the overall goal is for the agent to maximize the cumulative rewards we can incorporate the concept of return *G* which is the sum of the discounted rewards from the current time step

$$G=\overset{\infty }{\underset{k=0}{\sum }}\gamma^{k}R_{t+k+1}.$$ (4)

The discount factor *γ* influences the agent’s behaviour by balancing immediate rewards with long-term objectives. As the agent takes actions it receives rewards; the value of these rewards is responsible for shaping the policy or value function. It is possible for agents to find a way to exploit the reward function, where the policy may converge to undesirable behaviour that never the less returns high rewards. Furthermore, reward shaping should pay close attention to both environmental and system-specific constraints such as actuator saturation or limited power supplies. For instance, small rewards or penalties might be given to encourage the agent to also minimize actuator effort. *R*_*goal*_ also poses a divide by zero error when the agent has reached the goal. It is common in this case to give the agent a large conditional reward for completing the mission.

In EM applications not only the complexities of the robot itself, but also the operating environments are intrinsically challenging. Furthermore, the missions that the robots are trying to accomplish can be complex. For instance, if the robot is expected to find the shortest path, to cover an entire target area, or detect a specific target in a search and rescue (SAR) operation. While some robots may be expected to operate in the same region for every mission, observing the same lake on a daily basis, for example In such scenarios, while the area may remain the same and known to the robot, the conditions can vary dramatically across multiple different time scales. In other applications, such as fire-fighting or emergency SAR the agents may have to adapt to completely new and *a priori* unpredictable environments in every mission. This means that effective active EM systems should be able to adapt online to a wide range of conditions and environments. Using RL techniques is one way by which we can generalise the performance of an agent and extend the functionality to multiple different environments.

It should also be noted that any real active EM system, that is put into a real use case scenario, should be designed with great care towards the energy constraints. This is because the time scale of these missions can be long undefined prior to the mission start and safe refueling locations may be few and far between. For EM systems to be effective and worthwhile they need to bring long-term autonomous persistent monitoring to the region of interest Egerstedt et al. (2018). With current battery technology, the vast majority of systems will have to stop and recharge. This is a much bigger issue for some platforms than others, for example, small autonomous multi-rotor UAVs have a very limited flight time Azar et al. (2021). This constraint adds additional layers of complexity for traditional optimization that can be quite simply added to a reward function in RL, making it easier to consider from the beginning of the design process, perhaps at the cost of additional training time.

One EM example where RL and DRL have proved to be effective is the patrolling problem Luis et al. (2020). In this problem, the agent must cover an area effectively under time and energy constraints. Furthermore, this problem can be inhomogeneous (when some areas of the region of interest may be of more importance than others). Here the agent must learn to cover the target area completely while more regularly visiting some areas than others. In real situations, these areas of high importance will also change themselves meaning that no area can remain forgotten for too long as it may have become important as time passes. One can see the complexity of the problems that active EM can pose and how RL offers a high-level and intuitive design that leads to the desired behaviour through trial and error.

One final note on the application of RL to EM is that in environmental sciences it is standard practice to use simulations for predictions and research. As a result, there are many sophisticated and standard simulations which can generate data and model environmental processes. These simulations could be used to train RL and DRL agents for active EM. This potentially means developers of RL systems do not have to build their own simulations which, due to time and budget restrictions, could potentially over-simplify the environmental dynamics negatively impacting the performance of the RL agent. And so there is a potential area of technical contribution that can be made by porting these simulations to popular development platforms in robotics like Gazebo which is used commonly in conjunction with the Robot Operating System (ROS).

### 2.4 Reinforcement learning for multi-agent environmental monitoring systems

So far we have discussed RL in the context of single-agent systems. However, multi-agent robotic systems or robotic swarms are ideal solutions to many of the challenges imposed by active EM. Multi-agent systems are teams of autonomous agents that can cooperate on their movements, sensing and computations to achieve a common goal. Using multiple agents for active EM improves performance, time and energy efficiency and allows the system to cover wider areas. It also can add redundancy as there are multiple robots performing the same task. Designing multi-agent systems, with traditional methods, can be challenging as not only does each individual robot have to be able to perform in a challenging environment, but also the collective behaviour must also be able to adapt in real-time to *a priori* unknown disturbances and avoid inter-agent collisions and ultimately adaptive behaviour is preferable in changing environments Busoniu et al. (2008).

In the same way that RL and DRL can be used to encode complex behaviour onto single agents, multi-agent reinforcement learning (MARL) can be used to encode the behaviours at the level of the swarm Kouzeghar et al. (2023). In MARL, the agents coexist and interact with the same shared environment. In such scenarios, they must consider how to maximize not only their individual reward but also a collective reward. When you have multiple agents in a shared environment their goals might be shared or opposed. In systems where agents share common goals the system is known as cooperative while in systems where agents have opposing interests, the system is called competitive. In systems with multiple optimization objectives, there may be a mix of competitive and cooperative behaviours and the reward function should reflect these goals. Consider the example of a team of factory robots tasked with cooperatively lifting a block towards a goal. Each agent will receive an individual reward for its own actions such as approaching and touching the block. However, we must now also consider the *joint reward*, which is a reward that all agents receive for actions that accomplish the overall system goal: to lift the block. In this case, the joint reward will be given when the block moves towards the goal location. While agents may learn to cooperate organically, joint rewards can also be given to directly encourage cooperation and shorten training time. In this example, this might be a joint reward for synchronized lifting actions.

Like the systems themselves, MARL training can either be centralized or decentralized. In centralized training approaches agents share experience from previous training episodes. This gives agents the opportunity to learn from data they would not have access to during execution Lin et al. (2023). In this case, the agents will have a richer idea of the operation environment and can learn from other agent’s policies while in a fully decentralized training, agents still only learn from their own experience. It is worth noting that some MARL systems are simply composed of multiple single agents learning in the same environment but not sharing any training data.

### 2.5 Limitations of reinforcement learning

As discussed RL and DRL can be used to train robust and intelligent agents for a variety of important applications. However, these algorithms are not without their limitations. One of the biggest limiting factors for RL is that it is very sample-inefficient. Training agents to operate, even in the simplest system, can take hundreds of thousands of iterations Li et al. (2023). This is especially limiting in the field of robotics where agents will be deployed on physical systems. In many cases, it is near impossible to train agents in the practical domain. To avoid this issue it is common practice to develop simulations that mimic the real operation environment and train the agent there. But here lies another limitation of RL, model mismatch. This is the difference between the simulation and the true environment, these differences are always present and since one cannot simplify the real world, simulations must capture all of its most important elements. It is conceivable that agents trained in simulation will converge to an optimal policy but not behave optimally on the physical system due to this mismatch. This is especially important for EM and in much of the literature addressed in this, paper simulation environments are not comprehensive. More dynamic solutions are available for robots, for example, in Feiyu et al. (2024) gazebo is used to train agents or in Liu and Bai (2021) where a Geographic Information System was used to generate terrain data. An important step for EM is to bridge the gap between existing engineering and environmental simulations and tools allowing researchers to utilize the advantages of both platforms. Despite these limitations practical results have still been achieved with constrained simulations but more accurate training environments often mean better and more reliable results.

The increasing complexity of simulation naturally brings an increase in training time with each iteration taking longer to process. A common critique is that development of RL can be slow since some agents require days to train. Complex systems also require powerful computational resources. RL algorithms are also known to be very sensitive to training parameter values such as learning rate and discount factor and attempts at training may lead to no solutions. Tuning these parameters relate to the balance of exploration and exploitation. This refers to whether an agent should choose to try a new action in a given state (explore) or take an action which it knows will bring some reward (exploit). Exploring may shed light on new, more desirable behaviour, but too much, may lead to intractable convergence times or no convergence at all. How to handle this trade-off is still an open question in RL. An incorrect balance of exploration and exploitation can lead to the singularity problem which is when the agent’s policy or value function becomes unbounded or diverges during training.

For some applications of RL, one may encounter the issue of sparse rewards. The sparse reward problem refers to systems in which an agent may only receive rewards in certain scenarios. Like in collision avoidance, for example, if the agent does not meet obstacles often then it may not learn how to avoid them Hu et al. (2023). Sparse rewards might also be inherent problems of the operation environment themselves, such as in an underwater environment. Despite these limitations, RL and DRL are still promising solutions to real and complex problems, but researchers should be informed of these issues during development.

### 2.6 A general framework for active environmental monitoring problems

The active EM problem can be formulated as a Partially Observable Markov Decision Process (POMDP). A POMDP is a mathematical framework used to model decision-making problems, though unlike a normal Markov decision process (MDP) a POMDP does not assume the agent has access to the true underlying states. This is a useful practical assumption for real systems as EM robots have limited information about the underlying target phenomena and even their own states in practice. Since agents cannot be certain of the state of the underlying function, they maintain what is called, *a belief state*. The agent takes actions based on the current belief state. Each action moves the agent to a new state and the agent receives a reward based on this outcome. The goal of an active sensing agent solving a POMDP is to take actions that provide the most information and maximize the cumulative reward over time. One can change how much an agent prioritizes immediate reward vs. cumulative reward by leveraging a discount factor. It is common for RL problems to be modelled as an MDP or POMDP in the literature. Nevertheless, in this work, we propose a more general framework that also includes active sensing techniques as a special case. Table 1 shows how the components of a POMDP are comparable in RL and active sensing. To generalize further, we formulate a decentralized POMDP (Dec-POMDP) to include multi-agent systems reviewed in Section 2.4 as they become more and more popular. It is also common to formulate multi-agent systems as a Markov game as discussed in Littman (1994). The Dec-POMDP is a tuple that consists of:

• *N* is the number of agents (for a single agent system *N* = 1).
• S is the set of possible states ***s***_*t*_ of the environment.
• A is the set of joint actions such that $A^{m}=\left\{a_{1}^{m},\ldots ,a_{t}^{m}\right\}$ is the set of actions of agent *m*.
• Z is the set of joint observations such that $Z^{m}=\left\{z_{1}^{m},\ldots ,z_{t}^{m}\right\}$ is the set of observations of agent *m*.
• T:S×A×S→[0,1] is the transition probability function.
• R:S×A→R is the joint reward function.
• *γ*_*t*_ ∈ (0, 1] is a discount factor at time *t*.

**TABLE 1:** Table summarizing the how components of the POMDP compare between active sensing formulations and RL formulations.

POMDP	Reinforcement learning	Active sensing	Summary
State S	Represents the environment’s true information which cannot be directly observed by the agent	Represents the underlying characteristic of the environment or phenomena that the agent is observing	Both maintain a beleif state $B_{t}\left(st\right)$ . RL focuses on representing unobservable true state, while active sensing aims to understand observed characteristics to reduce uncertainty
Action A	Actions are taken by the agent to influence the environment based on the current belief state $B_{t}\left(st\right)$	Actions are the agent’s decisions on where to sample and collect data. Actions are typically selected to maximize information gained and/or reduce uncertainty in the belief state $B_{t}\left(st\right)$	RL actions influence environment directly, while active sensing actions aim to maximize information gain through data collection
Observation Z	Observations in RL provide partial information about the hidden state	Observations represent the data or measurements collected by the sensors	Typically directly comparable used to infer the underlying state S
Transition Function T:S×A×S	This is an internal model of the environment describing the probability of how the environmental states evolve from the current state to the next state under the influence of a specific action ***a***_*t*_	A model of the environment describing how it’s characteristics change when agents take a specific action ***a***_*t*_. Typically models the dynamics of what is being sensed	In RL, transition function models state evolution; in active sensing, it models environmental characteristics change
Reward Function R:S×A	A single scalar feedback value that determines how effective a given action was at the current time step. It is a high-level description of the agent’s desired outcomes	The value of information gain. Assigned based on how well the latest sensing action reduces uncertainty in the belief state	RL reward is more literal, it is intrinsic to learning and can incorporate the active sensing reward
Discount Factor *γ*_*t*_	A scalar factor that determines how much weight the agent is giving to the future long-term rewards compared to the immediate reward	A factor that balances between exploration of new unobserved regions and exploitation of data in the previously sensed regions with high level of information	RL balances future rewards against immediate, active sensing balances exploration against exploitation of existing information

Since it is assumed that an agent does not have access to ground truths about its state in a Dec-POMDP problem, a joint belief state is maintained which is the probability of being in a state given the history of all joint actions and observations:

$$B_{t}\left(s_{t}\right)=P\left(s_{t}|z_{1:t},a_{1:t}\right),$$ (5)

The joint action space A is described by all the actions an agent can take, the observation space Z is made up of all the possible observations an agent can make, and the transition function describes the probability of transitioning from the state ***s***_*t*_ to state ***s***_*t*+1_ when joint action ***a***_*t*_ is taken. The reward function is a type of information that measures the ‘goodness’ of performing a joint action ***a***_*t*_ in a given state ***s***_*t*_, the discount factor is used to decide how much to consider future rewards compared to immediate reward when taking the next action. It is worth noting that in model-based active sensing and RL, the agent can make a prediction of a future state based on its current belief and predicted observation ${\hat{z}}_{t+1}$

$$B_{t+1}\left(s_{t+1}\right)=P\left(s_{t+1}|{\hat{z}}_{t+1},a_{t+1},B_{t}\left(s_{t}\right)\right).$$ (6)

The goal of solving the Dec-POMDP is to find an optimal joint policy π* that maximizes the total reward. A single joint multi-agent policy could be given as:

$$\pi^{*}=\arg\underset{\pi }{\max}E\left[\overset{H}{\underset{t=0}{\sum }}\gamma_{t}R\left(s_{t},a_{t}\right)\right],$$ (7)

where *H* is the time horizon and the expectation is taken with respect to the probability of making a joint observation given the current belief state and joint action $P\left(z_{t+1}|\left(B_{t}\left(s_{t}\right),a_{t}\right)\right)$ .

## 3 Methodologies in active environmental monitoring

In EM there are a number of common methodologies used to achieve specific outcomes or incorporate essential required behaviour for an autonomous system which are addressed in this section. Specifically, we look at coverage, patrolling, path planning, collision avoidance, exploration, search and rescue, source seeking, source estimation and boundary tracking. In practice, these behaviours are often closely linked and may be used together on a single system. For example, a search and rescue system may need to use a coverage protocol to ensure no targets are missed within a search area. Thanks to the flexibility of RL it has been widely applied to each of these behaviours and the literature in this section is organized according to which methodology it is applied to.

The application of RL to these systems requires many design considerations. The learning agent needs to have access to information that allows it to learn how its actions influence the environment and how it can take subsequent actions to maximize reward. This requires careful design of a state-space and training environment that provides the needed information and is a reliable model of the true environment representing its features and constraints alike. The state space is often used as the input for the RL agent and thus the policy should be designed accordingly. This is also true of the action space which should reflect the environment and the demands of the physical system, a ground robot cannot change its altitude, for example. The state space is often used as the input for the RL agent and the action space is the output. This means that a compatible RL algorithm and policy should be chosen. There is a significant body of research in RL that combines different learning algorithms with different policies. In a recent example, the authors replaced the standard Q-table in Q-learning with a neural network that used a radial basis function to represent the policy Wang Y. et al. (2024). While the state space may contain information not directly accessible in the real environment, the observation space should only contain information that is feasible for the agent to access. For instance, in source estimation, the agent would not be able to observe the true state of the field, only discrete sensor readings, but in simulation training this ground truth is known. For some ground truths, that are only available in the simulation environment, can be strategically used to reward the agent during training. The reward function itself is designed in a way that reflects the overall goal of the system and considers the constraints of the operating environment. It is common to use different rewards under different conditions like using one reward to encourage exploration of new areas when the local proximity of the robot is free of obstacles but in the presence of obstacles reward the agent for successfully avoiding them. This strategy can also help elevate the problem of sparse rewards present in some applications and environments.

### 3.1 State representation in active environmental monitoring

A large portion of the literature in RL is based on model-free approaches as they do not have the issue of model mismatch and are generally quicker to train. However, it is often the case in EM that the goal of the system is to produce a model of the environment, and hence, there is a higher proportion of model-based RL approaches in EM literature. The model state S can include the environmental phenomena we are observing, the dynamics of the environment, the dynamic state of the robot and other agents in the team. The way that the model is represented depends on the specific nature of the system, the monitoring task and the RL algorithm.

In reality, environmental phenomena are continuous but attempting to model them exactly can make training difficult and slow. As a result, it is often the case that the state space is discreteized. In EM, this is often done via tile coding Padrao et al. (2022) in which the state space or environment is split into a 2D grid of tiles. In systems where there is a need to highlight specific information or features of the environment, one can use a relevance map. A relevance map is made by encoding these tiles with information that helps an agent make decisions. Tile encoding and image inputs are also viable for 3D state representations in active EM. For example, Liu and Bai (2021) uses DRL for coverage path planning and represents the 3D path of the agent as a 2D grey-scale image. This is achieved by what they term ‘the elevation compression method’ where the grey-scale value of each cell represents the altitude. Using feature extraction the CNN can successfully extract a 3D path from the grey-scale values. These types of state space representations are abstractions of the environment and are sometimes represented as an image, which is a concise way of presenting such information. Abstracting the environment to these discrete representations is good for reducing complexity but can limit performance to be optimal with respect to the abstracted environmental *representation* rather than the actual real-world environment.

As mentioned before, some research in RL has been applied to the patrolling problem. The patrolling problem in EM involves the agent revisiting the same locations periodically. Furthermore, in the real world, the application of patrolling is often inhomogeneous, meaning that some areas with a higher importance must be visited more frequently than others and the importance of the areas itself may also be variable over time. The environment in such problems can be represented as a relevance map. Luis et al. (2020) studied the case of monitoring the Ypacaraí Lake which is an essential source of drinking water and a popular tourist destination in Paraguay. The Ypacaraí Lake is a commonplace case study for the conservation of water resources. The Ypacaraí Lake is unfortunately suffering from an abnormal eutrophication process due to increased agriculture in the surrounding areas. Autonomous surface vehicles (ASVs) have been deployed and multiple different representations of the environment were considered. First, a model-free approach is used where the environment state S is just the position of the agents, then a binary relevance map that splits the lake into cells and colours visited cells as black and un-visited as white, which models a homogeneous patrol. Finally, a relevance map that colours cells a shade of grey which depends on the time since the last visit and their level of importance. The latter relevance map-based method leads to the best results. The state is passed to a convolutional neural network for DRL.

Relevance maps have also been used for patrolling with UAVs Piciarelli and Foresti (2019). In this work, the relevance map is also used to solve an inhomogeneous patrolling problem and the colouring grid represents the importance of areas. The relevance map is combined with a binary mask, which tells us which states have been visited, before being passed to a Convolutional Neural Network (CNN), which is used to extract features from images. UAVs that utilize tile-coded maps have also been useful in SAR applications Lu et al. (2022). The target area is represented as a grid of sub-areas. Each sub-area or ‘pixel’ can have 1 of 3 values: imaged, being imaged or un-imaged. The goal is to train a policy to image all areas. It is also worth noting that in many UAV applications, the navigation module of an autonomous system will have to consider no-fly zones. These are areas where it may not be legal or safe to fly a small UAV. Theile et al. (2020) develop a state representation as a 3-channel map where the channels describe no-fly zones, target zones and take-off and landing zones. RL has also been discussed for use in agricultural applications Binas et al. (2019). A similar environmental representation is used for agricultural applications in MARL Faryadi and Mohammadpour Velni (2021). Here the world is split into a ‘grid world’ where each grid point is a possible state. The goal of the study is to find free states, target states and states blocked with obstacles.

Not all model-based approaches use relevance maps. For example, Chen J. et al. (2020); Rückin et al. (2022); Viseras and Garcia (2019) use Gaussian Processes to represent the environmental phenomena. A Gaussian process (GP) is a probabilistic model often used in EM to represent spatial and spatio-temporal fields. GP regression allows agents to develop continuous predictions of a field and give variance values at each prediction point based on a set of discrete observations. Learning a GP as a model of the environment is different from a GP being used as a function approximate for the RL policy (in place of a neural network in DRL, for instance) and the reader should take care not to confuse the two applications Gadd et al. (2020). GPs have also been implemented in RL to improve exploration during training Zhao et al. (2024).

It should be clarified that not all systems in the literature that use some kind of representation of the environment are model-based. The classification depends strictly on how the RL algorithm learns. For example, in Placed and Castellanos (2020) DRL is used to make decisions in active Simultaneous Localization and Mapping (SLAM). The distinction here is that even though we have a DRL agent guiding the robot’s position for the SLAM process the inputs to the DNN are limited to the most recent laser scans, not the map produced by SLAM. Here the RL agent does not have any information retention about the model and cannot predict future observations based on previous ones.

### 3.2 Actions, observations and rewards in active environmental monitoring

As already discussed, it can be very difficult to hard code complex behaviour onto robotic systems and RL provides us with strategies for doing so. In EM and robotics, the nuances of the desired behaviour depend on the specifics of each problem but there are overall mission goals common across the literature. This section reviews the different methodologies used to achieve those mission goals. As seen in the previous section, there are multiple ways to represent the environment/agent state S , nonetheless, how a system can act to achieve its goals depends on its observations {Z} , action space {A} and reward function R . The main factor, driving the resultant behaviour of a system is the reward function. Ultimately, the principal aim of reward shaping is to guide training to be efficient and effective in producing the desired performance and optimal policy. Typically, rewards can be sparse or dense. A sparse reward function means the agent receives a reward only occasionally which can lead to long training times because agents need more experience to understand the implications of specific actions. Sparse rewards can be the result of an intentional design choice or of a sparse environment, like in deep-sea applications. Dense rewards are given more frequently and can produce more informative feedback on each action. While a well-designed dense reward function can speed up the training convergence and lead to good performance, it is worth noting that they can also impose the designer’s preconceived notions of optimal behaviour on the agent rather than allowing the agent to learn for itself. It is also important to find a good balance between an action space which is simple enough to allow the training to converge in a reasonable time frame and also complex enough to allow optimal behaviour.

#### 3.2.1 Coverage and patrolling

It is often the case in EM where agents will need to perform full area coverage. This is where a single agent or a team of *m* agents are tasked with covering an area completely under time or energy constraints. The coverage problem appears in a plethora of EM applications and is often implemented as a search step prior to other mission goals like source seeking or SAR which are described later. For example, in Wu et al. (2024) a coverage algorithm for multi-agent maritime SAR is proposed in which DRL is used to train agents to navigate to a grid location that has the highest probability of containing a target. This probability is based on novel drift simulations at a given time which predicts target trajectories and search boundaries that allow an agent to find targets thus facilitating their rescue. In real-world scenarios, the desired area to cover may not be defined *a priori*, like in the case of monitoring a wildfire, which can spread rapidly over time. Coverage also encapsulates patrolling which has been partly discussed in Section 3.1. A summary of RL-based coverage and the relevant literature is provided in Table 2. A large proportion of the areas we want to study with EM are boundless or undefined meaning that complete and exact coverage becomes difficult. Most readers are probably familiar with the travelling salesman problem where an agent, the salesman, must find the shortest path that visits every node once and then returns to the origin. Once the salesman has visited every node it can be said that the salesman has achieved coverage. The travelling salesman and coverage problems are generally NP-hard. That means as the problem scales up, the time needed to find an exact solution becomes infeasible Ai et al. (2021). This does not even account for the complex, dynamic, and partially observable nature of the environmental phenomenon aimed for monitoring. This is where RL is employed to learn the optimal coverage behaviour through interaction with the environment. Luis et al. (2021) compared the use of DRL techniques to evolutionary algorithms for the application of patrolling the Ypacaraí lake. It was found that DRL performs better when there is a higher number of solutions which is expected in EM applications. Evolutionary algorithms are also well suited to active EM for similar reasons to RL and the combination of both is trending research Zhang et al. (2023).

**TABLE 2:** Characteristics of reinforcement learning based coverage and patrol.

Characteristic	Description
Objective	Maximize area coverage and importance (patrolling)
State Representation	Environment states as grid cells, importance information
Action Space	Movement actions and sensing actions
Reward Function	Rewards for total coverage, visiting new areas, patrolling importance
Observation	Environmental sensing target
Challenges	Sparse rewards, large spaces and inhomogeneous patrolling
Citations	Pham et al. (2018); Faryadi and Mohammadpour Velni. (2021); Theile et al. (2020); Luis et al. (2020); Lu et al. (2022); Kouzehgar et al. (2020); Luis et al. (2021); Ai et al. (2021)

In Lu et al. (2022), they point out the limitation of the field of view for visual sensors used in SAR UAVs. In the case of a camera, at lower altitudes less of the target area will be in the frame and there is a need for an efficient coverage algorithm to make sure every section of the target area is scanned. In this work, the scanning mechanism is included in the discrete action space {A} (scan left or scan right) along with the movement actions (fly straight, turn left, turn right). A DRL agent is trained using Deep Q Learning (DQN) to fully cover the target area while minimizing the number of actions taken. To this end, movement actions are given a small penalty (negative reward) R as they only assist in the scanning of the target area but do not achieve it directly. There is also a penalty for when the UAV is not inside the target area. A reward is given proportional to the number of cells in a relevance map that have been scanned or are currently being scanned. A large reward is given if complete coverage occurs. Although this method was shown to improve the efficiency of UAV SAR coverage the system is too simplified to allow for true optimal behavior.

The coverage problem is also directly suited to multi-agent systems and it is easy to see how multiple agents with the possibility to collaborate can cover a given area more efficiently than a single-acting agent. Pham et al. (2018) proposed using MARL to design a distributed algorithm for coverage of an unknown field while minimizing overlapping scanned areas with other agents. The system is formulated as a Markov game, which is an extension of an MDP that includes a joint state space S and action space for multiple agents interacting with the same environment to optimize a joint policy. In this work, individual agents have a discrete action space A that allows them to pick one of 6 actions: north, east, south, west, up or down. The set of actions agents take is called the joint action $\left\{A^{m}\right\}$ . Agents must reach a consensus on the next joint action. To ensure coverage while minimizing overlapping, agents get an individual reward $R^{m}$ proportional to the number of cells they covered minus the number of overlapping cells. There is also a global reward applied to all agents, equally used to help train the converge problem faster.

Kouzehgar et al. (2020) proposed MARL for a structured approach to area coverage for a team of ocean buoys. They modify the Multi-Agent Deep Deterministic policy-gradient (MADDPG) reward function to intrinsically bring about the collective behaviour of the swarm. The reward $R^{m}$ depends on the state S of the swarm and thus, the reward itself has a collective behaviour. A positive reward is given based on the overlap between an ‘agent coverage polygon’ (a circle around the agent) and the ‘region polygon’ (the target area) and the total overlap which represents the total area covered. While the agents still receive some independent reward based on their actions the nature of this design is to reward the swarm as a whole for desirable *collective* behaviour rather than to encourage individual behaviour within the swarm.

#### 3.2.2 Path planning and collision avoidance

Path planning and collision avoidance are often solved under the same problem framework. This is because, in practical situations, obstacles are often the reason that advanced path-planning techniques are required. In EM, especially SAR or indoor applications, a robust method for detecting obstacles and planning trajectories that allow the robot to navigate around them safely is crucial. However, many standard methods of collision avoidance are designed specifically for static obstacles Choi et al. (2021). RL provides a good option as it can be trained on many different environments without having to hard code behaviour for each one making it easy to generalize to unseen environments. A summary of RL-based path planning and collision avoidance algorithms and the relevant literature is provided in Table 3.

**TABLE 3:** Characteristics of reinforcement learning based path planning and collision avoidance.

Characteristic	Description
Objective	Find safe, efficient paths and avoid collisions
State Representation	Environment states, possibly occupancy grid
Action Space	Movement actions or possible trajectories, sensing actions
Reward Function	Penalty for collisions, rewards for avoidance and goal reaching
Observation	Sensor data for obstacle detection (e.g., vision or LiDAR)
Challenges	High-dimensional states, real-time constraints and dynamic obstacles
Citations	Larsen et al. (2021); Popovic et al. (2020); Rückin et al. (2022); Chen et al. (2017); Yanes Luis et al. (2022); Choi et al. (2021); Woo and Kim. (2020)

On the other hand, for UAVs that operate at a high altitude, such as for terrain mapping or pollution monitoring, there are not likely to be many obstacles that an agent will encounter, especially in single robot systems where there are no inter-agent collisions. It is, however, especially important for UAVs to have efficient and reliable path-planning capabilities due to their restricted flight times. Due to the nature of environmental processes, autonomous UAVs need to be able to adapt in real time to the information they collect. This is known as informative path planning (IPP) and is a crucial step in active EM. Rückin et al. (2022) combined DRL with a Monte Carlo tree search to reduce the computational burden of the predictive planning. This is also useful in development during the simulation stage as comprehensive active sensing simulations can be expensive. They also address the issue that, in the current literature, the action space is typically 2D $A\subseteq R^{2}$ . This is because 3D action spaces $A\subseteq R^{3}$ are very large, making it difficult for training to converge. Nevertheless, this simplification does not make use of an aerial vehicle’s principal virtue which is their ability to move in three dimensions. To fully unlock the potential of autonomous aerial systems for EM, more work like this must be done to include 3D action spaces. The action space $A\subseteq R^{3}$ considered is a discrete set of possible measurement positions. The reward received by the RL agent depends on the reduction in predictive uncertainty of the environmental model and the cost of the action taken. The cost of the action is the flight time between measurement locations. The environmental model is a Gaussian Process which is updated using a Kalman filter Popovic et al. (2020). A Monte Carlo tree search is used to traverse the policy, which is represented as a convolutional neural network to decide the most information-rich actions. This is stopped when a budget or maximal search depth is reached. This tree search speeds up training and performance by removing the need to predict the informativeness of every possible action allowing for 3D action spaces. The authors showed their method performs better than multiple benchmarks.

Larsen et al. (2021) compared some state-of-the-art RL algorithms for navigating unknown environments on an ASV. The desired behaviour was efficient path planning and collision avoidance to reach a position goal. The algorithms tested were Proximal Policy Optimization PPO, Deep Deterministic Policy Gradient (DDPG), Twin Delayed DDPG (TD3) and Soft Actor-Critic (SAC). The performance for each algorithm was measured not only by the total reward but also by mission progress, collisions, time taken and cross-track error. The algorithms were all implemented using Python and StableBaselines which are discussed in Supplementary Material. The simulated operation environments were randomly generated in calm ocean settings with moving obstacles being introduced later, in more challenging environments. Agents were tasked with reaching the goal without colliding along the way. The reward function R punishes agents for colliding or being too close to obstacles and rewards them for progress towards the goal. After training, each agent was tested in environments of growing complexity. It was found that PPO produced agents that performed consistently across all environments in cases where the other agents did not. A repeat of these tests was run on agents trained with a simplified reward function that was more sparse. In this case, it was found that a sparse reward function stunted the performance in every case. While PPO still shows superior performance its generalisation capability is not nearly as good as the case under a denser reward. This work sets a good example for comparing RL algorithms in EM and demonstrates that more standardization across the literature would afford a more empirical consensus on the performance of state-of-the-art algorithms in different applications.

As mentioned UAVs are an ideal choice for EM since they can fly, but much of the research for autonomous UAV navigation focuses on 2D space since 3D spaces are more complex. In Wang J. et al. (2024) a 3D path-planning algorithm with collision avoidance is proposed. Collision avoidance is inspired by the International Regulations for Preventing Collisions at Sea (COLREGS). It uses four distinct collision avoidance behaviours, which are part of the discrete action space, to avoid collisions with an obstacle. The UAV will undergo collision avoidance behaviour when an obstacle is within a certain distance of the UAV. Using a spherical Artificial Potential Field (APF) that has 3 zones: safety zone, collision avoidance zone and mandatory collision avoidance zone, the UAV can choose what action to take. The authors, note that RL can be difficult to apply to collision avoidance due to sparse rewards, and solves this by using the different zones of the APF to design a dynamic and conditional reward that rewards an agent for approaching its 3D goal way-point when no obstacles are present in the collision zone. When an obstacle is detected the agent is rewarded for avoiding collisions by using the correct collision avoidance behaviours derived from COLREGS.

COLREGS is designed for maritime missions and applying it to robotic applications in bridges the gap to real-world implementation. COLREGS is also leveraged in Li et al. (2021) with DRL to perform collision avoidance for an ASV. The APF is also used to generate a dynamic reward function that eliminates the problem of sparse rewards. In this solution, DQN is implemented with a continuous action space that represents the heading for the agent. This system, which is designed to adhere to the mandatory COLREGS results, is only tested in numerical simulations and so does not take into account the complexity of the real world and the highly dynamic and variable ocean environment. Another example of using RL to train agents to follow COLREGS is given in Wen et al. (2023). Here, a multi-agent team of ASVs are trained to avoid collisions with other agents in the system, environmental obstacles and other ships. An action evaluation network is pre-trained using a large data set of real, pre-recorded COLREGS avoidance trajectories. This is combined with an action selection network for cooperative path planning. The action selection network is trained on individual agents using DRL. The reward function rewards agents for successfully avoiding obstacles in such a way that aligns with COLREGS.

As mentioned, multi-agent systems hold massive potential and are the key to the future success of autonomous active sensing in EM. Cooperation for multiple agents adds complexity to both path planning and collision avoidance. One may choose to use RL in such systems as it allows agents to learn coordinated policies that may be complicated and time-consuming to hard code. Yanes Luis et al. (2022) uses DQN with prioritised experience replay to develop a multi-agent framework for IPP. The proposed method is tested and applied to the Peralta et al. (2020). They also use their framework to address the credit assignment problem Mao et al. (2020). This is a critical challenge in RL and refers to the difficulty of determining which actions or decisions taken by an agent in the past, contributes to a particular reward or outcome.

#### 3.2.3 Autonomous exploration and SAR

Exploration is different to the coverage problem as it is concerned with autonomously gathering information about its environment to discover unknown areas often with the aim of achieving other tasks. Coverage on the other hand aims to visit every point of a given area. These two are often combined if the aim is to cover an *unknown* area Chen et al. (2021) but sometimes other goals are also combined with exploration. Autonomous environment exploration is required for applications like SAR Zuluaga et al. (2018). SAR is one of the most popular applications of RL in environmental monitoring. Providing effective solutions has great value in reducing human fatalities following disasters where traditional resources can become stretched. Research has been conducted into both indoor GPS-denied SAR and outdoor SAR with and without GPS localization. Most of these applications note the need for sophisticated intelligence in these agents as they often need to navigate previously unknown, dynamic, and cluttered environments and identify victims within them. A summary of RL-based exploration and SAR along with the relevant literature is provided in Tables 4 and 5 respectively.

**TABLE 4:** Characteristics of reinforcement learning based exploration.

Characteristic	Description
Objective	Discover unknown environment
State Representation	Environmental features, grid cells or frontiers
Action Space	Movement actions, sensing actions
Reward Function	Rewards for successful exploration, penalty for revisiting areas
Observation	Sensor data for map building or data collection
Challenges	Balancing exploration and exploitation, stopping conditions
Citations	Niroui et al. (2019); Hu et al. (2020); Chen et al. (Chen et al., 2020a; Chen et al., 2020b); Maciel-Pearson et al. (2019)

**TABLE 5:** Characteristics of reinforcement learning based search and rescue.

Characteristic	Description
Objective	Locate and assist or recover targets
State Representation	Environmental features, grid cells, target locations
Action Space	Movement actions, alarm/communicate, sensing actions
Reward Function	Rewards for finding targets, exploration, safe navigation
Observation	Sensor data for navigation or target detection (e.g., vision or LiDAR)
Challenges	Handling dynamic environments, long horizons, target detection
Citations	Zuluaga et al. (2018); Sampedro Pérez et al. (2019); Peake et al. (2020); Kulkarni et al. (2020); Ai et al. (2021)

It can be argued that due to the unpredictability of SAR environments, such systems should not solely rely on a good GPS signal and redundancy is required in their localization system. GPS-denied localization is often achieved through SLAM, especially in indoor environments. This uses a vision sensor like a camera or LiDAR to iteratively map the surroundings and localize the robot within them. Active SLAM refers to systems that use SLAM data to autonomously navigate their environment to facilitate more accurate measurements of the environment Placed et al. (2023). Active SLAM can also fit under the umbrella of active sensing and the Dec-POMDP discussed in section 2.6. SLAM can be combined with RL agents to achieve active SLAM in cluttered environments useful for SAR applications. Placed and Castellanos (2020) use a trained DDQN and Dueling double deep Q Learning (D3QN) policy as the decision-making module in active SLAM. SAR missions would also typically involve some methods for detecting victims. In Sampedro Pérez et al. (2019) 2D LiDAR SLAM is used for localization while a DDPG agent is used to perform image-based visual servoing. This is where image features are used as control signals to direct the sensing agent towards a target. Both high-quality simulation and experimental results demonstrate that RL can be used in this system to produce effective solutions for image-based visual servoing in cluttered environments. This paper also demonstrates that RL systems can be robust to model mismatch between simulation and reality. The agents were trained largely through simulations but still performed well in real-world environments.

During SAR exploration it is important to make sure that all of the region of interest is searched. One popular companion of SLAM for indoor exploration is called ‘frontier exploration’. A frontier is defined as the border between mapped and unmapped areas that are not known to be an obstacle wall or boundary. The hypothesis is that agents can achieve complete exploration by visiting frontiers until there are none left. The standard method of choosing which frontiers to visit is either breadth-first (nearest frontiers) or depth-first (most distant frontiers). Niroui et al. (2019) proposes using an Asynchronous Advantageous Actor-Critic (A3C) DRL agent as an alternative method of frontier selection to improve the operation time of a frontier exploration-based system. The agent aims to maximize the information gained along a robot’s path of exploration. Compared with several traditional exploration techniques implemented on a physical robot, the authors prove the superiority of their method in exploring the target area in a smaller total distance.

Cluttered environments also exist in outdoor applications and agents must also deal with potentially difficult weather conditions. An important consideration for the actual deployment of these algorithms is that different environments may demand different physical UAVs with varying payload capacities and different sensors. Robustness to such potential hardware changes is one of the key requirements in developing these algorithms. Maciel-Pearson et al. (2019) proposed an algorithm that can be used on any UAV with any camera for autonomous exploration of outdoor environments. They tested their algorithm in a simulated forest under different conditions. The DRL agent is trained directly to explore a region of interest and is penalized for collisions, revisiting areas, and leaving the search area. A double state input S is utilized to learn collision avoidance from raw sensor data Z , and complete exploration from navigational history and an environmental model. Outdoor environments are often difficult to deal with due to size. Peake et al. (2020) developed a SAR UAV to search a large area in a wilderness by splitting the area into unique regions on a quadrant tree. Each quadrant is assigned a probability distribution indicating that it currently contains a victim. A DDQN-trained policy is then used to select which of these quadrants should be visited next and generate an optimal flight trajectory across segments A that maximizes information gain along the way. A second DRL agent trained using Advantageous Actor-Critic (A2C) is used to allow the agent to explore the target quadrant more freely to find a potential victim via a continuous action space $A_{\mathrm{cont}}$ . This combination of discrete and continuous action spaces is a clever way of negating the drawbacks of each option. The DDQN agent interacts with a large area. A common and effective strategy for exploring large areas while saving resources and ensuring convergence of the training is to discretize the area. This can lead to a loss of optimally for the true continuous environment. However, within the quadrant, which is a small cell of the full environment, a continuous action space is used which allows more dynamic movement and better exploration of the original continuous environment, thus reducing some disadvantages of a discretized environment.

Like all applications in EM, using multiple cooperative agents can improve exploration performance. The benefits of cooperation are especially true for SAR as these applications are time-critical. A common technique for allocating search areas is to split the target region into Voronoi partitions which can be either dynamic or static. Hu et al. (2020) combines dynamic Voronoi partitions for exploration with a DRL collision avoidance algorithm to allow agents to safely explore their partitions. The assignment of Voronoi partitions is an effective way to stop agents from colliding with each other, however, navigating in a cluttered environment with obstacles demands full online collision avoidance. DRL has proved to be an effective solution for using raw sensor data for collision avoidance Long et al. (2018); Chen et al. (2017).

As described in Section 2.6, active sensing tasks mostly follow a generalized design philosophy of adaptive information gathering. Sensing decisions are made based on collected data and/or a belief model of the environment or target phenomenon Chen F. et al. (2020). Viseras and Garcia (2019) proposed an algorithm based on multi-agent DRL to make an algorithmic framework that can be applied to different information-gathering tasks without changing the underlying algorithm. To incorporate the needs of different applications both free and model-based approaches are proposed. The base algorithm, named Deep-IG, is a multi-agent extension of the A3C algorithm. The shared reward function is dual objective, simultaneously minimizing both the total mission time and the normalized root mean squared error in predicting the time-invariant physical process. Agents are also penalized for inter-agent collisions. The action space A , is made up of discrete high-level robot movement commands. Deep-IG is shown to work across different physical platforms without changing algorithmic parameters. Experimental data is collected with 4 quadrotors for a terrain mapping application and ground robots are used for magnetic field mapping. Generalized reliable algorithms are valuable for EM applications as every deployment situation is different. Efforts such as this pave the way for widespread deployment as the bespoke algorithms and hardware designs for an intractable amount of potential monitoring applications are unrealistic, especially nowadays that climate change demands a quick answer.

#### 3.2.4 Source seeking and boundary tracking

Source seeking is useful in many areas of EM, whether it is finding the location of a gas or radiation source, finding a wildfire in a forest, or finding a victim in a SAR scenario. In practice, the source concentrations can be very low and the data collected can be highly noisy, especially when far away from the source. In such cases, RL agents can become robust to high-noise environments with a sufficient number of training episodes. In general, concentration can be considered to be exponentially decaying as the distance from the sources increases. This means it is common for reward functions R in source-seeking applications to be directly related to the observed concentration values Z . Some systems are also concerned with estimating the properties of the target phenomenon whose source is being located, this is termed source term estimation (STE). Table 6 contains a summary of RL-based source-seeking algorithms and the relevant literature.

**TABLE 6:** Characteristics of reinforcement learning based source seeking and estimation.

Characteristic	Description
Objective	Locate the source of phenomenon
State Representation	Environmental features, source model grid cells
Action Space	Movement actions, sensing actions
Reward Function	Rewards for locating source
Observation	Intensity/Concentration values of environmental target
Challenges	Noisy and sparse information, dynamic sources, initial search phase
Citations	Duisterhof et al. (2021); Li et al. (2023); Viseras and Garcia. (2019)

Source seeking via DRL has been demonstrated on a nano quadrotor with very highly constrained computational resources. Using a cheap commercially available and common processor, Duisterhof et al. (2021) demonstrated a source-seeking DRL agent that is robust to high noise and shows consistent performance across several real and simulated environments. A neural network with only two hidden layers was trained to locate a source from concentration measurements. Systems such as these demonstrate a true potential for RL in EM. The main contribution of this work is how affordable and disposable the nano quadrotors are and their ability to operate in environments that may pose a risk to human life. This system uses a discrete action space A , made up of high-level commands and a decoupled flight controller capable of translating these into actuator commands.

Li et al. (2023) propose an active RL algorithm. Active RL means there is information from the current state of the sensing process guiding the decisions and hence training of the agent. Here, the information metric is based on the maximum entropy sampling principle. This method combines both model-free and model-based approaches in RL into one algorithm based on recent promising results from similar approaches Nagabandi et al. (2018); Ostrovski et al. (2021). A logarithmic reward function is used to account for the exponential nature of concentration decay over physical space. In this system, they used an RL agent to optimize the dual objectives of the source seeking and source estimation; the agent must locate the source and maintain knowledge of the spatial concentration distribution. To achieve this, keeping a balance between the exploration of new areas and the exploitation of the previously collected information is necessary. The trade-off between exploration and exploitation is intrinsic to RL and active sensing alike. In RL specifically, this is manipulated by changing between future and immediate rewards using a discount factor *γ* defined for each agent. This trade-off is itself also a common optimization objective in time-constraint systems, where the agent becomes more exploitative as the mission progresses. The contribution of this work is the use of active exploration within RL which is shown to increase sample efficiency.

Gas source localization applications are often time sensitive as dangerous gasses have the potential to do harm quickly and good STE is required to coordinate an appropriate response. RL approaches for source seeking and estimation tend to direct agents by means of a discrete grid which can be limiting when applied to practical scenarios. Park et al. (2022) leverages DRL to find the optimal search path using a continuous action space. While the proposed action space does contain a continuous heading allowing for more diverse movement the limitations of a discrete action space are not totally elevated since the robot moves a fixed distance per step. For instance Zhang et al. (2023) combines a continuous heading with a continuous speed command. To allow for quick estimation of the gas distribution a particle filter is used and a Gaussian mixture model is used to extract features that make for suitable inputs to an instance of DDPG. They implement a gated recurrent unit (GRU) into their DNN that allows for memory of previous states and better STE. They verify the effectiveness of this choice by comparing the learning performance with and without the GRU.

Another area of research in which active EM can be applied is concerned with controlling environmental conditions in sensitive environments like artificial habitats or laboratories. While much of this research is concerned with novel control algorithms some have proposed using mobile sensors to complement this process don (Bing et al., 2019); Jin et al. (2018); Xiang et al. (2013). Another example of where environmental conditions must be closely monitored is in a space habitat like that of the International Space Station. In Guo et al. (2021) they propose using moving sensors for temperature monitoring and fire detection in a space habitat called the Active Environmental Monitoring and Anomaly Search System. They implement a dynamic value iteration policy (DVI) to solve the problem which is modelled as a MDP. The performance of the DVI policy is measured against numerous other benchmarks. The DVI policy approaches the “jump policy” in anomaly detection time. This is a promising result since the implemented jump policy is not physically viable as it does not consider the continuous space a sensor would have to move through and assumes that it can “jump” to the next measurement position. The system is also extended to a distributed multi-agent system by implementing multiple signally trained agents. The authors point out that in confined and sensitive atmospheres it is important to consider how much benefit can be extracted by adding more sensors so that the optimal trade-off between anomaly detection time and number of sensors can be found. However, this has limited implications as MARL and cooperatively trained agents are not considered.

An interesting idea for using source seeking in an SAR application is presented in Kulkarni et al. (2020). A Q-learning RL agent is trained to locate victims via radio frequency signals of a device possessed by the victim. This, for example, could be a smartphone or a smartwatch. Using a discrete action space A , the objective is to find the source or the victim, in the shortest possible time. The reward function R , is based on the received signal strength (RSS) Z , which has also been demonstrated for localisation in GPS-denied environments. The system is tested using ray tracing software to provide a better and more realistic simulation than simple numerical models. This helps the RL agent to perform upon deployment as it was trained in an environment more similar to the true environment.

In wildfire monitoring systems, manned aerial missions are often used to track the boundary and spread of the fire. This helps make decisions on where to use fire suppressants and where to remove fuel for fire containment. It can also be used to motivate and prepare evacuations if the fire is heading towards settlements. Viseras et al. (2021) proposed single and multi-agent DRL systems to locate and track the boundary of a wildfire for both multi-rotor and fixed-wing type UAVs. Two MARL approaches, i.e., multiple single-trained agents (MSTA) and a value decomposition network VDN, are proposed. In both cases, the agents are trained by a joint reward function R . They found that both proposed algorithms outperform the benchmarks. More specifically, VDN tends to perform better towards the end of an episode when the fire behaviour is more complex and coordination between UAVs is more important, whereas MSTA provides a better performance early on when the main goal is finding the fire. They also found MSTA to be more stable and scales better when there are more than three agents. Table 7 contains a summary of how RL boundary tracking algorithms are posed.

**TABLE 7:** Characteristics of reinforcement learning based boundary tracking.

Characteristic	Description
Objective	Track boundaries of phenomenon
State Representation	Environmental features, object boundaries
Action Space	Movement actions, sensing actions
Reward Function	Rewards for accurate boundary tracking, penalties for errors
Observation	Sensor data for measuring phenomenon (e.g., vision sensor or intensity/concentration measurements)
Challenges	Noisy or ambiguous boundaries, initial search phase
Citations	Julian and Kochenderfer, (2019); Viseras et al. (2021)

Boundary tracking can also be applied to UAV systems that are equipped with fire fighting capabilities, with the aim of containing the spread of a fire by applying suppressants at the boundary of the fire. This is the goal of the work in Ostrovski et al. (2021). Here, a multi-agent fire-containing system of UAVs is trained using multi-agent DQN (MADQN) in which each agent acts independently but can learn from a pool of shared agent experience which helps to accelerate training. The authors encourage the cooperation of agents by removing any extra effect if agents apply fire suppressants to the same location. This research includes both sensing and communication constraints and a bespoke forest fire simulation. Since agents are limited to communicating with only their neighbours the system is decentralized and scalable which is very valuable for EM applications.

In the mentioned works that discuss boundary tracking, however, the simulations are entirely numerical and thus make a large number of assumptions about the operation environment. While the results in these papers are promising and further demonstrate the benefit of applying RL to active EM systems there is a large step between simulations like this and practical implementation. This is a trend across all the EM applications discussed but boundary tracking has a significant lack of practical results in the RL sphere.

### 3.3 Open challenges

Many of the system behaviours covered in this work are themselves well explored in the literature and the consensus is that autonomous systems are a big part of the future of EM. However, there is a shortage of research applying these behaviours to specific environmental domains, especially within the field of RL. Most of the existing efforts are simulation-based. These simulations are often greatly simplified versions of the true environments or focus on one specific element like a temperature field or wind model. There is potential for a large body of interesting research where robotic and RL-based EM systems are developed by making use of existing ecological simulations. Simulation is a large part of ecological research and collaboration between the two disciplines can help accelerate the implementation of the important systems discussed in this paper. Simulations that utilize these advanced environmental models would be much closer to the true operation environment reducing model mismatch and accelerating work towards the practical domain. RL offers an easy first step to this unification as training is done offline and data can be collected beforehand. One example of early efforts in this space is given in Liu and Bai (2021) where a geographic information system (GIS) is used as the DRL training environment to provide the terrain data for a simulated UAV. This allows them to consider areas that may be obstructed from certain perspectives which are not considered in 2D terrain-based simulations. Besides, in EM there is a lack of practical testing and much more effort is required in the practical domain to start to build the road to actual implementations of these systems. Practical results are crucial for development and at the current stage, any practical testing offers immense value.

One of the biggest criticisms of RL is its sample inefficiency. As RL is better understood and solutions improve sample-efficiency will get better but effort is needed to address this directly. One promising area is the combination of model-based and model-free approaches Pong et al. (2020); Chebotar et al. (2017). This has been applied to EM applications in Li et al. (2023) to improve STE. These approaches aim to extract the benefits of both model-free RL that does not suffer from model mismatch and the learning efficiency of model-based approaches. Another way to improve sample efficiency is using long-term prediction Tutsoy (2022); Doerr et al. (2017). Long-term prediction involves estimating the future states of the environment, the rewards associated with those states, and how the agent’s actions will influence the system over multiple time steps. Accurate long-term prediction allows agents to take more optimal actions earlier in the training process. It is especially useful when the consequences of actions are not immediately apparent.

Another open research area is the use of multi-agent reinforcement learning scenarios. The benefits of multi-agent systems are clear, especially for tasks like EM which can have such a large and diverse scope. It is apparent from the literature covered in this paper that most research in the RL for EM work is focused on single-agent implementations and the existing MARL approaches are rudimentary compared to the demands for a full operational system. There is an *extreme* lack of practical results for MARL despite promising simulation results and the clear potential of these systems. In light of these challenges, we offer a summary of some of the potential research areas that would help accelerate the development of systems capable of full-time deployment.

• Simulations that include more complete environmental models for specific environmental domains.
• MARL-based EM system research.
• Both single and multi-agent practical implementations at real sites of interest.

## 4 Real-world deployment challenges

There is a lack of practical application of these methodologies in the field of active EM. It is crucial that more practical testing is carried out and documented in the literature for fully autonomous EM systems to be realized. As stated, the environments are often difficult to operate in and each specific environment will bring its own challenges. Furthermore, some of these systems will have to operate in multiple different environments. Thus the characteristics of these environments must be closely studied. In the literature, it is common to refer to environments as being either indoor or outdoor. Indoor environments are generally smaller, more cluttered and GPS-denied making navigation and localization challenging. On the other hand, outdoor environments are often much bigger, less sparsely populated, GPS enabled and have large variations due to changing light and weather conditions. That being said this does not hold for all outdoor environments, for example, active EM systems designed for operation in a forest will have to deal with unreliable GPS and a high density of potential obstacles which is more consistent with indoor environments. A simplified comparison of some environments of interest is given in Table 8. One benefit of RL is that it can be trained under these considerations by utilizing multiple training environments and iterations of similar environments. It can learn to perform well under large variations and respond well under unknown conditions.

**TABLE 8:** Comparison of environments where active EM may be applied.

	Rural	Urban	Lake/River	Deep-sea	Sub-terranean	Nuclear	Farming	Forest
GPS	Good	Medium	Good	None	None	None	Good	Bad
Scale	Large	Large	Predefined	Large	Medium	Small	Large	Large
Pre-defined Area	No	Maybe	Yes	No	Maybe	Yes	Yes	No
Weather Sensitivity	High	Medium	Medium	Medium	Low	None	High	Medium
Robot-Type	UAV, AGV	UAV	UAV, ASV, AUV	AUV	UAV, AGV	UAV, AGV	UAV, AGV	UAV, AGV
Commu-nication	Good	Good	Good	Bad	Bad	Good	Good	Medium
Obstacles	Few	Lots	Few	Few	Medium	Lots	Medium	Lots
Light	Variable	Variable	Variable	Dark	Dark	Light	Variable	Varible (Darker)
Charging Potential	Medium	Good	Good	Medium	Bad	Bad	Good	Bad

Another limiting factor for the practical application of active EM systems is the energy constraints of the platform. For example, UAVs have a very short flight time. Active EM systems will inevitably have to stop and refuel regularly. This requires safe and accessible locations to install charging points or places for the system to remain idle and charge passively via a utility such as on-board solar panels. In some environments, safe charging locations may be hard to find. For example, it is the case in volcanic monitoring that these charging stations will often have to be far away from the survey region as the areas close to the volcano are usually inaccessible and dangerous. This again highlights the need for such systems to have an optimal energy performance but also sheds light on the infrastructure needed for long-term practical application Egerstedt et al. (2018); Notomista and Egerstedt (2021). These environments may also pose challenges for maintenance and retrieval. In some environments, such as nuclear decommissioning, where the robot is in an environment that is potentially lethal to humans, retrieving the robot may be impossible. This means that the robots need to be very reliable and robust to the challenges of their environment. It also demands that they can communicate their findings without physical collection. For certain extreme environments like deep-sea or subterranean environments, wireless communication is a big challenge.

It is often the case that AUVs often periodically resurface to both recharge, communicate their data and connect with other agents. In underwater multi-agent systems like this, RL could be used to predict the position of other agents. This is a valuable corrective action as When the team is submerged it is unable to communicate and apply corrections until the communication is available again. Or RL has been used for processing acoustic signals which are a method of underwater communication Rahmati et al. (2019). MARL can also be used to teach agents how to cooperate without or with very limited communication Zhang and Lesser (2013). Since in RL, the training is done before deployment, MARL systems can take advantage of training agents based on other agents’ training data that would not be available during run time. Having a good simulation environment can teach agents to cooperate without any communication when they are deployed.

Underwater environments pose lots of unique challenges that may have simple solutions in other domains Shruthi and Kavitha (2022). Further to communication restrictions, the environment is also GPS-denied and non-variant, making localization extremely challenging. The uniform nature of the underwater environment also makes rewards very sparse and thus the learning efficiency and rates are often unfavourable. One option is to use a human trainer. This can either be using human-based examples to produce a reward or to have a human providing a reward based on good or bad behaviour. Zhang et al. (2020) proposed a DQN-based system that learns a path following control policy from a human-given reward and a pre-defined environmental reward. The use of human reward is helpful here as the underwater operation environment means that the environmental rewards can be highly sparse. This can ultimately lead to the system not converging to an optimal policy.

Certain survey areas of interest may be predefined before the mission starts, the size and shape of a lake, for example, while others may change constantly. For example, if we consider the problem of monitoring the plume of a volcano, the position of the plume with respect to the source, will change largely based on the strength and direction of the wind on a given day. It is a waste of time and energy resources to survey areas where the plume does not exist. This changing area of interest is common to many EM problems. The extreme case of this is when the survey area is completely unknown like in an urban SAR response to a natural disaster. Some environments where active EM is required are very isotropic and lack any clear landmarks, appearing very uniform to the agent. This is especially true for underwater, deep-sea applications. This is a big challenge for successful localization and navigation especially when GPS is also not an option. It is also a challenge for RL agents as the nature of the environment means that the rewards will be very sparse.

It is also the case that applying these systems in certain environments can be potentially dangerous to humans. For example, using UAVs in urban areas runs the risk of a malfunction causing injury to pedestrians or property damage. To remove or minimize this risk in busy cities there should be no-fly zones. It may also be the case that these will change, based on the time of the day or current events. Some social challenges come along with some environments, especially highly populated, urban areas. For example, if autonomous ground vehicles were deployed for air-quality monitoring in cities, there is a potential for theft and vandalism. Thus, not only do they have to be safe for operation around the general public but they must have strong security features and be physically robust. Citizens may also dislike the idea of autonomous systems being used due to a lack of trust in the technology. Finding ways to introduce these systems in ways that are least offensive to the public is a real concern Çetin et al. (2022).

## 5 Conclusion

In this work, we have reviewed the application of RL and DRL to robotic active sensing in EM. The escalating climate crisis has highlighted the need for comprehensive EM solutions. Challenging, unknown and dynamic environments are ubiquitous to EM applications and intelligent and adaptive solutions are needed for autonomous and persistent monitoring that we can rely on. We have discussed RL as a method of encoding complex behaviours onto single and multiple agents for active EM. To provide a unifying framework we have utilized Dec-POMDPs to frame these systems as an active sensing pursuit and to unify them with traditional active sensing approaches. We discuss methods of representing environments as in EM there is more of a demand for model learning than in other areas of RL. The main body of this review is separated into common mission goals and agent behaviours that are useful in EM. We conclude this review by discussing and comparing the spectrum of the most widely used technical development platforms available for researchers in the hopes of streamlining further development and collaborating on a framework for standardization.
