# Supplementary material for: A survey on autonomous environmental monitoring approaches: towards unifying active sensing and reinforcement learning
Source: Front Robot AI. 2024 Mar 12;11:1336612. doi: 10.3389/frobt.2024.1336612 (PMC10964253; doi:10.3389/frobt.2024.1336612)
Supplement: Supplementary file 1 [file DataSheet1.pdf]

Supplementary Material

1 DEVELOPMENT PLATFORMS FOR RL

In this section, we will review some of the most popular development platforms for RL. Python has gained significant popularity for RL development in recent years. This is largely due to the rich ecosystem of libraries and frameworks. It is also clear that across reinforcement learning there is a lack of information on the specifics of technical implementation and a lack of standardization. The platforms described in this section can provide researchers with a well-supported set of compatible tools for streamlining development and standardizing benchmarking and performance metrics Henderson et al. (2019) The features of these platforms are summarized in Table S1.

Platform	Open Source	Language	Pre-Built Environments	Flexibility	Community Support
TensorFlow	Yes	Python	Yes	High	Large
PyTorch	Yes	Python	Yes	High	Large
Gymnasium	Yes	Python	Yes	Moderate	Moderate
Stablebaselines3	Yes	Python	Yes	High	Moderate
PettingZoo	Yes	Python	Yes	Moderate	Moderate
MATLAB RL toolbox	No	MATLAB	Yes	Moderate	Moderate

Table S1. Feature comparison of reinforcement learning development platforms.

1.1 TensorFlow

TensorFlow is a comprehensive open-source ML framework maintained by Google. In TensorFlow users create static data flow graphs that describe how data moves Abadi et al. (2015). The nodes of the graphs represent mathematical operations and the edges represent the flow of data. By manipulating these graphs users are provided a high amount of flexibility when building machine learning models. Keras is a popular tool for the implementation of Neural Networks (NN) with TensorFlow as it aims to be a minimalist and intuitive framework and can integrate with pre-built TensorFlow models to facilitate deep learning. TensorFlow comes with a steeper learning curve than other platforms, especially for users new to machine learning models. However, there is a large community of support and a pool of free learning resources due to its widespread popularity.

1.2 PyTorch

While Tensorflow has been widely recognized as an industry standard, PyTorch, which is a newer tool, is often considered more user-friendly and 'pythonic' Paszke et al. (2019). It is good for quickly prototyping models and is gaining popularity in the research community. While the graphs in TensorFlow are static, PyTorch uses dynamic graphs that are executed during run-time. While this can be useful for debugging execution time can be longer for bigger systems.PyTorch has its own in-built deep learning framework that provides the equivalent functionality to Keras in TensorFlow.

1.3 Gymnasium

OpenAI's GYM is a popular open-source platform for RL as it is fully compatible with both TensorFlow and PyTorch Brockman et al. (2016). It provides pre-built benchmark-type environments that allow users

to easily test their learning algorithm, some example environments are showing in Figure S1. Tools like GYM are not only useful to help streamline RL development but also offer means of standardization, a necessity that is lacking in RL and DRL research. In GYM's early life maintenance and development were poor and as of October 2022, OpenAI GYM is being maintained by the Farama Foundation whose aims are to provide standardization and long-term maintenance of RL libraries[1]. The future of Gym will take place under the name of Gymnasium (although the names are often used interchangeably). The Farama Foundation is coordinating future development with other big RL projects like StableBaselines which we discuss in this section. The environments provided by Gymnasium have defined actions, observations and rewards. Different environments allow users to explore different action and observation spaces both continuous and discrete providing 2D visual renders of the environment. GYM also provides the tools for the user to build their own environments. It can also interface with the Robot Operating System (ROS) making it popular for robotics and extending the application to three-dimensional dynamics.

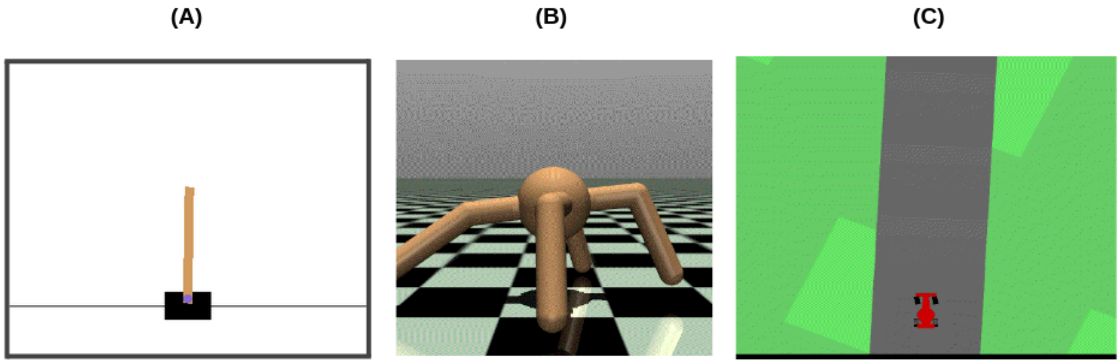

Figure S1. Examples of Gymnasium training environments (A) "Cart Pole" (B) "Ant" (C) "Car Racing".

1.4 StableBaselines and StableBaselines3

StableBaselines is a set of implemented and up-to-date RL algorithms that is an expansion upon the original baselines by OpenAI Hill et al. (2018). Originally made in TensorFlow, a more recent package StableBaselines3 was realised for PyTorch Raffin et al. (2021). This is a comprehensive and easy-to-use package that works well with OpenAI GYM. StableBaselines and StableBaselines3 allow users to easily implement state-of-the-art RL algorithms easily making it a strong platform for research with quick prototypes and intuitive use. This package if updated regularly can help researchers standardize benchmarking with state-of-the-art algorithms.

1.5 PettingZoo

PettingZoo Terry et al. (2021) is another project from the Farama Foundation that provides a simple and easy-to-use Python API. It provides similar benchmark environments and environment-building tools but for MARL, some example environments are shown in Figure S2. It provides both turn-based and parallel learning environments. Since MARL is a relatively new research area it provides a good standardization for bench-marking from the get-go. In terms of robotic applications, there aren't any standard environments that allow the training of a robot-like agent. However, there are a number of relevant third-party environments

[1] https://farama.org/Announcing-The-Farama-Foundation

available through the petting zoo online documentation. For example, Crazy-RL provides an environment for RL application on crazyflie drones [2].

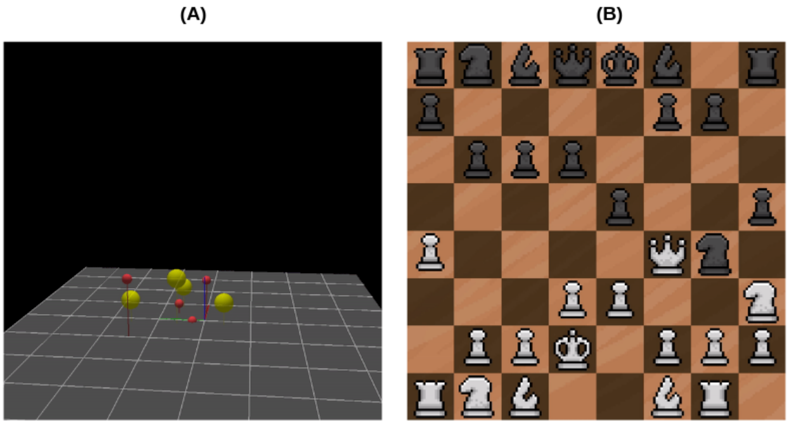

Figure S2. Examples of Petting zoo environments (A) ”Crazy-RL” (3rd party) (B) ”Chess”.

1.6 MATLAB

MATLAB has long been a standard tool in engineering research and it offers a well-developed RL Toolbox [3]. The toolbox includes neural networks for representing policies (DRL) and a number of standard RL algorithms available to train these policies. MATLAB is generally considered easy to learn due to its simplicity, comprehensive documentation and active professional support. This makes MATLAB’s RL Toolbox a good place for people to learn the foundations of RL. Furthermore, through the ONNX model format, the MATLAB toolbox is compatible with TensorFlow Keras and PyTorch, allowing users to take advantage of both MATLAB and pythonicc platforms. MATLAB also has Simulink which is a graphical user-friendly environment that can make the design process more intuitive. However, MATLAB doesn’t have the same wide scope that Python has to offer in terms of community support, ongoing projects, extra libraries and ease of implementation with many other platforms. MATLAB is also a closed-source and proprietary which may make it void for the individual.

REFERENCES

Abadi, M., Agarwal, A., Barham, P., Brevdo, E., Chen, Z., Citro, C., et al. (2015). TensorFlow: Large-scale machine learning on heterogeneous systems Software available from tensorflow.org

Brockman, G., Cheung, V., Pettersson, L., Schneider, J., Schulman, J., Tang, J., et al. (2016). Openai gym

Henderson, P., Islam, R., Bachman, P., Pineau, J., Precup, D., and Meger, D. (2019). Deep reinforcement learning that matters

Hill, A., Raffin, A., Ernestus, M., Gleave, A., Kanervisto, A., Traore, R., et al. (2018). Stable baselines. *GitHub repository*

Paszke, A., Gross, S., Massa, F., Lerer, A., Bradbury, J., Chanan, G., et al. (2019). Pytorch: An imperative style, high-performance deep learning library

Raffin, A., Hill, A., Gleave, A., Kanervisto, A., Ernestus, M., and Dormann, N. (2021). Stable-baselines3: Reliable reinforcement learning implementations. *Journal of Machine Learning Research* 22, 1–8

[2] *https://github.com/ffelten/CrazyRL/tree/main*

[3] *https://uk.mathworks.com/products/reinforcement − learning.html*

Terry, J., Black, B., Grammel, N., Jayakumar, M., Hari, A., Sullivan, R., et al. (2021). Pettingzoo: Gym for multi-agent reinforcement learning. In *Advances in Neural Information Processing Systems*, eds. M. Ranzato, A. Beygelzimer, Y. Dauphin, P. Liang, and J. W. Vaughan (Curran Associates, Inc.), vol. 34, 15032–15043
